# The Rising Significance of Soft Skills in Postgraduate Education: Nurturing Well-Rounded Professionals for the Modern World

**DOI:** 10.1590/0037-8682-0245-2023

**Published:** 2023-09-22

**Authors:** Lucindo José Quintans-Júnior, Dalmo Correia, Paulo Ricardo Martins-Filho

**Affiliations:** 1 Universidade Federal de Sergipe, Laboratório de Neurociências e Ensaios Farmacológicos, São Cristóvão, SE, Brasil.; 2 Universidade Federal de Sergipe, Departamento de Medicina, Aracaju, SE, Brasil.; 3 Universidade Federal de Sergipe, Laboratório de Patologia Investigativa, Aracaju, SE, Brasil.

In recent years, the development of socio-emotional skills and personal relationships in the workplace has gained prominence. With advancements in artificial intelligence and automation, many technical skills traditionally valued in the job market are becoming obsolete, and soft skills are emerging as crucial competencies. Although soft skills can be defined as "interpersonal" or "behavioral" abilities essential for success in professional and personal contexts, it is necessary to reflect on their importance in the current context of postgraduate training in human resources and research.

Given the growing importance of mental health in an increasingly complex and fast-paced world, improving the ability to manage emotions, developing refined sociability, and adopting a more thoughtful approach to decision-making have become top priorities for individuals seeking outstanding results. This is especially applicable to postgraduate students, emphasizing two key aspects: the extent to which these students can cultivate their highly valued skills and the importance of this process as a distinct study area.

Soft skills represent a set of competencies that individuals can develop to effectively manage their emotions, plan and achieve goals, and improve interpersonal relationships^1^. These skills encompass emotional intelligence, leadership, effective communication, teamwork, and adaptability, empowering individuals to navigate the challenges they encounter throughout their academic and professional endeavors^2^. Currently, proficiency in soft skills is considered a prerequisite for success in the job market and represents a significant asset for those entering postgraduate programs. The journey of an aspiring scholar is replete with challenges: demanding excellence in research, active participation in academic events, publication in scholarly journals, meeting coursework obligations, and adhering to strict deadlines for reports, dissertations, and theses. Furthermore, the capacity to articulate intricate concepts and inspire others assumes significance in research presentations as it facilitates securing funding for projects and garnering support for proposals.

In the emerging field of soft skills in the realm of study and research, we are witnessing a burgeoning area that has captured the attention of business communities. Companies seek individuals with social, emotional, and behavioral competencies, allowing them to thrive in team collaboration, leadership, and adaptability to changes^3^. Consequently, academic researchers are increasingly drawing on the study of soft skills to address this demand and investigate how such strategies contribute to innovation and entrepreneurship. In an era characterized by fierce competition in the job market and the imperative to capture consumer attention, acquiring effective mechanisms that enhance performance is paramount, potentially becoming the defining factor for the success of any given endeavor.

In postgraduate education, the importance of soft skills has become increasingly evident and is actively sought by researchers and universities. In today's contemporary world, professionals must possess solid technical skills and the ability to collaborate effectively in group settings, even under high-stress conditions, tight deadlines, and situations with limited infrastructure and financial resources. Furthermore, the quality and competitiveness of these products have reached unprecedented levels. Unfortunately, these pressures and other factors such as career devaluation and low salaries, have led to demotivation and concern for mental health disorders, including anxiety and depression. Although soft skills alone may not offer a definitive solution to these challenges, they play a crucial role in preparing a society that is becoming less emotionally resilient, mitigating interpersonal relationship issues within complex interdisciplinary teams and ultimately contributing to the system's overall productivity.

Universities worldwide are actively engaged in discourses surrounding the importance of non-technical proficiency, including soft skills. They are committed to fostering the development of students and researchers in this regard^4^. Thus, building on our achievements in other domains, we aspire to become the cornerstone of these crucial discussions. Notably, educational institutions are responsible for preparing students to operate in various contexts and realities. They play a central role in developing key competencies related to soft skills, known as the "4Cs": creativity, critical thinking, collaboration, and communication^5^. As the job market undergoes transformation and consumer profiles evolve, universities must remain steadfast in their commitment to uphold the standards of education, research, extension programs, and social inclusivity. By strongly emphasizing sustainable development and improving the quality of life, universities can effectively respond to the changing landscape and contribute to the holistic growth of individuals and communities.

Moreover, public universities in developing countries continue to play a crucial role in nurturing well-rounded professionals with the skills necessary to tackle labor market challenges and make significant contributions to society. However, it is essential to re-evaluate existing national postgraduate education models, moving beyond their technocratic tendencies and embracing a holistic approach that recognizes students as multifaceted individuals. This necessitates the inclusion of disciplines such as anthropology, sociology, and philosophy, which have become increasingly vital in the contexts of today. This underscores the need for a comprehensive reassessment of the current postgraduate education system to align its priorities with graduates’ profiles and roles in the contemporary world. By adopting this approach, universities can better equip students with the diverse set of skills and perspectives required to address the complex challenges of our time and contribute meaningfully to society, as well as create mechanisms to monitor the success of their graduates in the job market and life, and whether their initiatives have been successful.
